# Energization of the Ring Current by Substorms

**DOI:** 10.1029/2018JA025766

**Published:** 2018-10-08

**Authors:** J. K. Sandhu, I. J. Rae, M. P. Freeman, C. Forsyth, M. Gkioulidou, G. D. Reeves, H. E. Spence, C. M. Jackman, M. M. Lam

**Affiliations:** ^1^ Department of Space and Climate Physics, Mullard Space Science Laboratory University College London London UK; ^2^ British Antarctic Survey Cambridge UK; ^3^ Applied Physics Laboratory Johns Hopkins University Baltimore MD USA; ^4^ Los Alamos National Laboratory Los Alamos NM USA; ^5^ Institute for the Study of Earth, Oceans, and Space University of New Hampshire Durham NH USA; ^6^ Department of Physics and Astronomy University of Southampton Southampton UK

**Keywords:** ring current, substorms, magnetosphere, RBSPICE, HOPE, Van Allen Probes

## Abstract

The substorm process releases large amounts of energy into the magnetospheric system, although where the energy is transferred to and how it is partitioned remains an open question. In this study, we address whether the substorm process contributes a significant amount of energy to the ring current. The ring current is a highly variable region, and understanding the energization processes provides valuable insight into how substorm‐ring current coupling may contribute to the generation of storm conditions and provide a source of energy for wave driving. In order to quantify the energy input into the ring current during the substorm process, we analyze Radiation Belt Storm Probes Ion Composition Experiment and Helium Oxygen Proton Electron ion flux measurements for H^+^, O^+^, and He^+^. The energy content of the ring current is estimated and binned spatially for L and magnetic local time. The results are combined with an independently derived substorm event list to perform a statistical analysis of variations in the ring current energy content with substorm phase. We show that the ring current energy is significantly higher in the expansion phase compared to the growth phase, with the energy enhancement persisting into the substorm recovery phase. The characteristics of the energy enhancement suggest the injection of energized ions from the tail plasma sheet following substorm onset. The local time variations indicate a loss of energetic H^+^ ions in the afternoon sector, likely due to wave‐particle interactions. Overall, we find that the average energy input into the ring current is ∼9% of the previously reported energy released during substorms.

## Key Points

- This study explored the spatial variations of the average ring current energy content for H+, O+, and He+ ions
- The ring current energy content increases following substorm onset with strong spatial dependences
- Approximately 9% of the energy released in a typical substorm is transferred to the ring current

## 1 Introduction

Substorms manifest as large scale, global reconfigurations of the terrestrial magnetosphere and a redistribution of energy within the system. The process has traditionally been defined by the occurrence of three separate phases forming a sequence: the growth phase, the expansion phase, and the recovery phase (e.g., Akasofu, 1968; Baker et al., 1996; McPherron, 1970; Russell & McPherron, 1973). The substorm growth phase occurs as a result of an enhanced low‐latitude dayside reconnection rate, usually initiated by a southward turning of the Interplanetary Magnetic Field (IMF), concurrent with a comparably small nightside reconnection rate (Milan et al., 2007). Open flux accumulates in the magnetotail, and energy is stored in the highly stretched magnetic field lines. Closely following substorm onset, which marks the initiation of the expansion phase, there is a sudden enhancement in the nightside reconnection rate. Explosive nightside reconnection rapidly closes the open flux in the magnetotail, releasing large amounts of energy and heating the plasma sheet (e.g., Forsyth et al., 2014; Huang et al., 1992). The expansion phase is followed by the recovery phase, where the nightside reconnection rate gradually reduces, and the magnetosphere returns to the average configuration. However, if the IMF remains southward, energy input continues to occur and the recovery phase may coincide with the growth phase of a succeeding substorm. The magnetospheric substorm is a highly complex process, displaying significant variability, and there are several open questions surrounding the substorm phenomenon. This paper addresses how the large amount of energy released at substorm onset is partitioned, specifically understanding the input of energy into the ring current.

Energization of the ring current is a key factor in the occurrence of a geomagnetic storm, and therefore, understanding the role of the substorm process in enhancing the ring current and its contribution to the occurrence of storms is of high importance. The substorm process can energize the ring current through several processes. For a detailed review we refer to Daglis (2006), but we briefly summarize here. During substorms, the combination of an enhanced background convective electric field with strong substorm induced impulsive electric fields have been suggested to aid in the injection of plasma deep into the magnetosphere and provide the ring current conditions necessary for geomagnetic storms (Daglis et al., 2004; Delcourt, 2002; Fok et al., 1999; Ganushkina et al., 2005; Lui, 2001; Metallinou et al., 2006; Reeves & Henderson, 2001; Reeves et al., 2003; Wolf et al., 1997; Wygant et al., 1998). Furthermore, substorms are associated with enhanced ionospheric outflow (Daglis et al., 1994; Daglis & Axford, 1996), which results in an increased plasma sheet density (Nosé et al., 2005). The convection of the high density plasma into the inner magnetosphere increases the ring current density and has been shown to be an important parameter in the generation of storms (Daglis, 2006). Alternatively, it has been suggested that the intensification of upward field‐aligned currents may also energize the ring current (Sun & Akasofu, 2000).

The occurrence of a series of multiple substorms is thought to be an effective means of energizing the ring current (Kamide, 1992), through recurrent heating of plasma originating in the magnetotail. The plasma is recirculated between the tail and inner magnetosphere as the field is alternately stretched during the growth phase and dipolarized in the expansion phase. This repeatedly accelerates the ions adiabatically by Fermi and betatron acceleration, resulting in the formation of a high‐energy tail in the ion distribution function (Daglis et al., 1999).

In this study, we examine how the total energy content of the ring current varies during the substorm process, providing insight into the role of substorms in the efficiency of energy transport and the occurrence of geomagnetic storms.

## 2 Data

This study employs observations obtained from the Van Allen probes (Mauk et al., 2013). This mission composes of two identically instrumented spacecraft, probe A and probe B, which have an orbital configuration with a perigee of ∼600 km altitude, an apogee of 5.8 R_E_ geocentric radial distance, and an inclination of 10°. The orbital period of the spacecraft is 9 hr, and the precession of the apogee provides sampling of all local times in less than 2 years. The low inclination spacecraft provides suitable measurements of the ring current region over all local times.

The Radiation Belt Storm Probes Ion Composition Experiment (RBSPICE) instrument onboard the Van Allen probes is a time‐of‐flight versus total energy particle analyzer that is designed to observe the ring current plasma population (Mitchell et al., 2013) and measures the particle distributions for H^+^, O^+^, and He^+^ ions. The Level 3 data products are used here, which provide omnidirectional particle flux measurements for H^+^ ions in the energy range of 50–660 keV, O^+^ ions in the energy range of 120–990 keV, and He^+^ ions in the energy range of 60–980 keV. The energy ranges of the instrument are highly suited to observe the ring current population, which is dominated by ions with energies of tens to a few hundred keV (Daglis et al., 1999). In this study, we use the omnidirectional flux measurements from probe A from 2012 to 2017.

Previous work has shown that although protons with energies between 100 and 300 keV are the dominant contribution to the ring current energy content during quiet times, significant fluxes of low energy protons are present in this region and therefore this population requires consideration (Daglis et al., 1999; Milillo et al., 2001). Furthermore, O^+^ ions can provide a substantial contribution at times, particularly during storm time conditions (Gloeckler et al., 1985; Hamilton et al., 1988; Krimigis et al., 1985; Williams, 1981; Zhao et al., 2015). Therefore, we have incorporated low energy H^+^ and O^+^ observations into this analysis. Measurements of omnidirectional H^+^ and O^+^ flux for the energy range of 1 eV to 50 keV from the Helium Oxygen Proton Electron (HOPE) instrument of the Radiation Belt Storm Probes‐Energetic Particle, Composition, and Thermal plasma suite (Spence et al., 2013) onboard the Van Allen probes are used (Funsten et al., 2013). Similarly to the RBSPICE data set, we use the Level 3 data product from probe A from 2012 to 2017. Previous work indicates, through cross‐calibrations with other instruments, that the HOPE instrument underestimates particle fluxes and therefore we multiply all HOPE fluxes by a factor of 3 in this study (Menz et al., 2016; Zhao et al., 2015). It is noted here that the multiplication factor introduces a possible source of error for the results associated with the HOPE data, as it is later shown in this study that the substorm‐associated variations occur on a smaller scale than the factor of 3 multiplication. However, due to the thorough cross‐calibration with other instruments, the HOPE fluxes remain a reliable measure of the low energy ion fluxes and should not represent a large source of uncertainty in our statistical results.

To summarize, we use observations from both the HOPE and RBSPICE instruments to provide coverage over a range of energies. It is highlighted that the energy coverage of O^+^ is not complete as there are no available observations provided by the particle instruments onboard the Van Allen probes between 50 and 120 keV. Therefore, the derived energy content values should be viewed as a lower limit that does not account for additional contributions from ions outside the energy ranges considered here.

## 3 Measuring Ring Current Energy Content Using Spacecraft Observations

Geomagnetic ring current indices, specifically the Dst index and the SYM‐H index, suggest a simple and effective route to estimating the total energy contained in the ring current. These indices are derived from ground magnetometer observations of the global variations in the horizontal field close to the magnetic equator. The stations used are located at latitudes that map to the ring current region (∼25° latitude) and cover a range of local times (Iyemori, 1990; Sugiura & Poros, 1964; Sugiura & Kamei, 1991).

The magnitude of the field perturbation can be related to the total energy of the ring current particles using the Dessler‐Parker‐Sckopke (DPS) relationship (Dessler & Parker, 1959; Sckopke, 1966). This approach assumes a dipolar magnetic field configuration and that the magnetic field depression measured by the Dst or SYM‐H index is solely due to the perturbation associated with the ring current. Due to the near‐continuous coverage over a substantial time period of the Dst index and the SYM‐H index, the use of the ring current indices pose an ideal method to measure variations in the ring current over substorms. However, previous work has shown that other current systems, such as the magnetopause, ground, and tail currents (referring to the definitions of Ganushkina et al., 2015), also contribute to the observed ring current indices (e.g., Alexeev et al., 1996; Asikainen et al., 2010; Burton et al., 1975; Campbell, 1973, 1996, 2004; Greenspan & Hamilton, 2000; Liemohn & Kozyra, 2003; Turner et al., 2000; Zhao et al., 2015). Although attempts have been made to account for additional contributions, such through the use of the corrected Dst* index (Burton et al., 1975) to account for the magnetopause current system, substorm‐associated variations are still not fully understood. In particular, the tail current system has been identified to be a significant contribution to the observed ring current indices during the various phases of the substorm process (Belova & Maltskv, 1994; Ohtani et al., 2001; Siscoe & Petschek, 1997; Turner et al., 2000).

Furthermore, analysis of the assumptions made by the DPS has demonstrated additional drawbacks of using this technique to quantitatively estimate the ring current energy content. In particular, the assumption of linear magnetic field distortions (Hoffman & Brachen, 1967; Lackner, 1970; Sozou & Windle, 1969a, 1969b), the implications of plasma inflow from the nightside plasma sheet (McPherron, 2013; Siscoe & Petschek, 1997), and the effects of a nonzero plasma pressure at the outer boundary of the integration volume (Liemohn, 2003) have been assessed.

**Figure 1:**
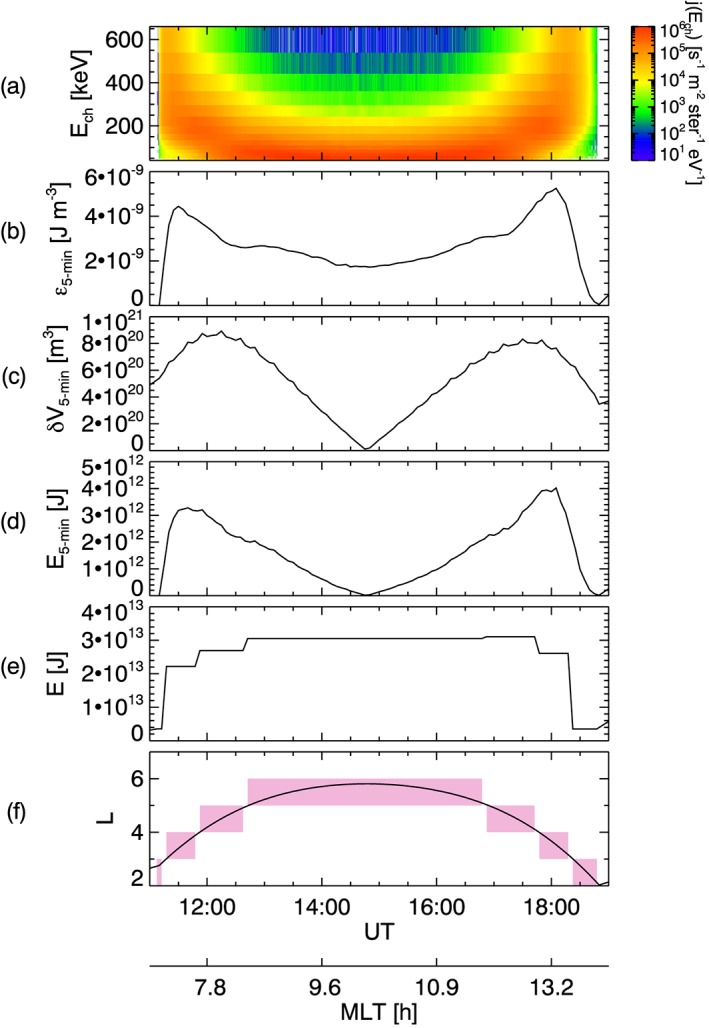
(a) Radiation Belt Storm Probes Ion Composition Experiment/Van Allen observations of omnidirectional proton flux, j(E_ch_), [s^−1^·m^−2^·ster^−1^·eV^−1^] plotted as a function of time (UT) and the instrument energy channels, E_ch_, [keV] during an apogee pass on 06 February 2016. Using 5‐min time bins, calculations of (b) partial energy density, ϵ_5‐min_, [J·m^−3^], (c) volume enclosed by the field lines crossed, δ V, [m^3^], and (d) partial energy content, E_5‐min_, [J] are shown. The total energy content, E, [J] is calculated for L‐MLT bins of L bin width 1 and MLT bin width 6 hr and shown in panel (e). For reference, panel (f) shows the spacecraft L value, with the pink shaded regions indicating the L bins that the spacecraft is traversing, during the pass. The MLT of the spacecraft during the pass is also indicated at the bottom of the figure. MLT = magnetic local time.

Alternative approaches include the use of energetic neutral atom measurements (Jorgensen et al., 1997), or as used in this study, estimating the energy content using in situ particle observations of the omnidirectional particle intensity for a range of energy channels, as used by Gkioulidou et al. (2016). The method employed by Gkioulidou et al. (2016) is now briefly described, where Figure 1 provides an illustrative example of the procedure. Figure 1 shows proton data from the RBSPICE instrument onboard Van Allen Probe A for an apogee pass on 06 February 2016. Figure 1a shows measurements of the omnidirectional flux, j(E_ch_), for each energy, E_ch_, of the instrument channels.

For a given time, the partial energy density, *ϵ*, can be calculated from the omnidirectional ion flux, *j*(*E*_ch_), using

(1) $$\epsilon =\sum_{E_{\text{ch}}}2\pi \sqrt{2E_{\text{ch}}m}j(E_{\text{ch}})\Delta E_{\text{ch}},$$

where Δ*E*_ch_ is the energy channel bin width and *m* is the ion mass. Figure 1b shows the partial energy density, *ϵ*_5‐min_, calculated using equation (1) and summing over all energy channels, where the values are averaged into 5‐min time bins.

The partial energy density, *ϵ*_5‐min_, for a given time bin is then multiplied by the volume of the region, *δ* *V*(*L*), to provide the partial energy contained within that volume, *E*_5‐min_. This is expressed as

(2) $$E_{\text{5‐min}}=\epsilon_{\text{5‐min}}\mathrm{\delta V}(L)=\epsilon_{\text{5‐min}}\left(V(L_{\max})-V(L_{\min})\right).$$

The volume is defined as *δ* *V*(*L*) = *V*(*L*_max_) − *V*(*L*_min_), where *L*_max_ and *L*_min_ are the maximum and minimum values, respectively, of the spacecraft within the 5‐min time period. The total volume occupied by the magnetic field within an *L* value, *V*(*L*), is

(3) $$V(L)=\left[\frac{64\pi }{105}R_{E}^{3}L^{3}\right]\left[\frac{B_{\text{dip}}}{B}\right]\left[\frac{24}{\Delta \text{MLT}}\right],$$

where *R*_*E*_ is an Earth radii (1 Earth Radii = 1*R*_*E*_ = 6,372 km). Equation (3) takes a dipolar magnetic field model and scales it by the factor ^*B*^_dip_/_*B*_, where *B*_dip_ is the magnetic field strength for a dipolar magnetic field model at the spacecraft position and *B* is the observed magnetic field strength as measured by the Electric and Magnetic Field Instrument Suite and Integrated Science instrument (Kletzing et al., 2013) onboard Van Allen probe A. This is done to account for the difference between the observed magnetic field and the dipolar magnetic field model and is an attempt to more accurately estimate the volume enclosed by the field lines at the given *L* value for the time of measurement. This approach was chosen as current empirical magnetospheric magnetic field models (e.g., Tsyganenko, 1995, 2002; Tsyganenko & Sitnov, 2005, 2007) are not able to provide an accurate description of the global magnetic field during the active and dynamic substorm times (Nishimura et al., 2011; Tsyganenko, 2013). Therefore, we use the scaled dipole as there is no suitably accurate alternative. Furthermore, equation (3) includes a magnetic local time (MLT) scaling factor (^24^/_ΔMLT_), which restricts the volume being considered to a segment of the region confined within the *L* value. Specifically, the volume extends only over a defined MLT bin width, ΔMLT, where an MLT bin width of 6 hr is used here. Using equation (3), the volume of the region sampled by the spacecraft during each 5‐min interval, *δ* *L*, is estimated and shown in Figure 1c. It can be seen that *δ* *V* has an orbital dependence. Figure 1c shows that the volume sampled in a 5‐min interval is notably smaller when close to apogee, as the spacecraft is traveling slower and therefore crosses through a smaller range of *L*.

Using the energy density (*ϵ*_5‐min_ shown in Figure 1b) with the volume estimation (*δ* *V* shown in Figure 1c), equation (2) allows the energy contained within the defined region to be estimated for the 5‐min sampling period. Figure 1d shows the calculated energy values for each 5‐min time bin, *E*_5‐min_. As a result of the *δ* *V* orbital dependence, *E*_5‐min_ also possesses an orbital dependence. With decreased spacecraft speed, *δ* *V* is lower and consequently *E*_5‐min_ is also lower (see equation (2). The effect of the orbital dependence is accounted for in the subsequent step.

In order to estimate the total energy contained within a spatial *L*‐MLT bin for the given data set, *E*, the energy values are summed as the spacecraft passes through the range of *L* values encompassed by the bin. This can be expressed as

(4) $$E=\left[\sum_{\Delta L}E_{\text{5‐min}}\right]\left[\frac{\Delta L}{\sum_{\Delta L}\mathrm{\delta L}}\right],$$

where Δ*L* is a defined *L* bin width. For the example shown in Figure 1, the energy values, *E*, for a *L* bin of width 1 are shown in panel (e). The values are plotted over the time taken to traverse the *L* bin, where Figure 1f shows the *L* values for reference. The scaling factor shown in equation (4) ( ${}^{\Delta L}{/}_{\sum_{\Delta L}\mathrm{\delta L}}$) is included to account for different trajectories through the spatial bin (e.g., for a apogee pass where the spacecraft would traverse a combined *L* value larger than the spatial bin width or for a partial pass through a spatial bin). The energy is divided by a sum over *δ* *L* ,the *L* distance traversed by the spacecraft during each 5‐min period. This essentially provides energy per unit *L*. The energy is then multiplied by the *L* bin width, Δ*L*.

It is noted here that equation (4) accounts for the orbital bias in *E*_5‐min_. Although *E*_5‐min_ is decreased with decreased spacecraft speed, the number of 5‐min samples obtained during an *L*‐MLT bin traversal is increased. As a consequence, the resulting energy content value obtained, *E*, correctly relates to the required volume extended by the bin.

The advantage of a technique based on spacecraft observations, as opposed to indirect estimates using ring current indices, is that spatial variations in ring current energy can be explored. Furthermore, the energy contained by different ion species can be assessed through the use of compositional ion intensity measurements.

## 4 Analysis of RBSPICE and HOPE Data

**Figure 2:**
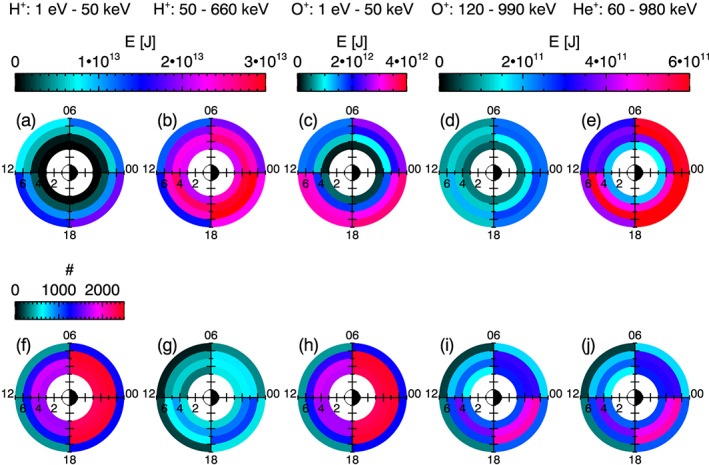
(a)–(e) shows the median energy, E, [J] for each L ‐ MLT bin, which is represented by the color and is plotted at the bin's location in the L‐MLT domain. Each panel corresponds to a different ion data set, as labeled. Using the same format, (f)–(j) shows the corresponding number of spacecraft passes, or equivalently the number of energy values, in each L ‐ MLT bin. MLT = magnetic local time.

The method described in section 3 is applied to both the RBSPICE and HOPE data sets in order to provide measurements of the ring current energy. The ring current energy was calculated using an *L* bin width of 1 and an MLT bin width of 6 hr. By binning the spacecraft passes for *L* and MLT, the spatial distribution of ring current energy can be identified and explored. Figures 2a–2e show the spatial distribution of the median ring current energy, plotted in the *L*‐MLT domain. Each panel corresponds to a different ion data set (1 eV–50 keV H^+^ ions, 50–660 keV H^+^ ions, 1 eV–50 keV O^+^ ions, 120–990 keV O^+^ ions, and 60–980 keV He^+^ ions) as labeled. The corresponding number of spacecraft passes through each bin are shown in Figures 2f–2j and indicate good statistical coverage for all bins. The results shown in Figure 2 include all measurements provided by the full data sets. We note that these typical ring current conditions are expected to be dominated by quiet periods of activity. In order to estimate the statistical error of the median values shown in Figures 2a–2e, the standard error of the energy measurements in each *L*‐MLT bin have been calculated. The results are shown in the supporting information Figure S1 for reference. Overall, the standard error of the median is orders of magnitudes smaller than the median values, and it can be assumed that the median values are sufficiently representative of the ring current.

Figures 2a–2e show that the majority of the ring current energy is contributed by the 50–660 keV protons. Variations in the energy content with local time are also apparent, where values are peaked in the 18–24 MLT sector for all ion data sets. We note that the energies calculated for *L*‐MLT bins at higher *L* values (e.g., comparing energies at *L* ∼ 6 to *L* ∼ 4) show higher energy values. This is a result of the varying bin volume. The volume enclosed by a *L*‐MLT bin with increasing *L* would also increase because the MLT bin width is kept constant. This restricts any analysis of how energy varies with *L*, but the variations with MLT can be assessed.

**Figure 3:**
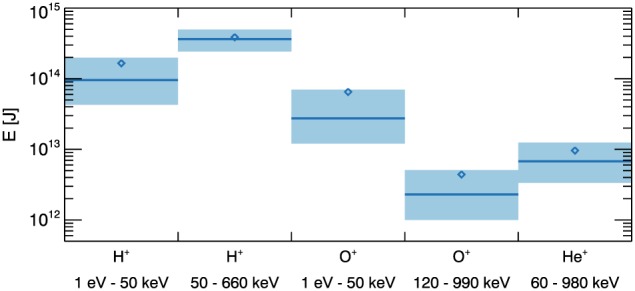
The sum of median energy, E, [J] values over all L‐magnetic local time bins for each ion data set, as labeled, are shown by the blue solid lines. The sum of the mean values is indicated by the blue diamonds. The corresponding sum of the lower quartile values and upper quartile values over the L‐magnetic local time bins are represented by the lower and upper limits of the blue shaded regions.

In order to estimate the total energy contributed for each ion data set, the median energies are summed over all *L* ‐ MLT bins. This is computed for the mean energies as well. To understand the spread of the values, the lower quartiles and upper quartiles are also summed over all bins. The results are shown in Figure 3, where the blue line corresponds to the median energy value, the blue diamond corresponds to the mean energy value, and the light blue shaded region corresponds to the extent of the lower quartile to upper quartile values. Similarly to Figure 2, the energies are shown for each ion data set.

As identified from Figure 2, Figure 3 also demonstrates that the 50–660 keV protons are the dominant contribution to the ring current. This result is in good agreement with previous studies, where Krimigis et al. (1985) reported that the protons within the energy range of ∼100–300 keV represent the main contributor to the total ring current energy. Figure 2 also provides an insight into the distribution of the energy values, where the 50–660 keV protons show an approximately coincident mean and median energy value, typical of a normally shaped distribution. In contrast, the other data sets demonstrate a skewed energy distribution toward higher energies.

The global energy content of the average ring current can be estimated from the values shown in Figure 3 by summing the median values over all ion data sets. This provides a typical energy content of 5.0 × 10^14^ J for the ring current. This value fits in well with previous observations of the energy content (Gkioulidou et al., 2016) and provides a reliable estimate of the average energy content of the ring current.

### 4.1 Dependency on Substorm Phase

To examine how the energy content of the ring current varies during a substorm, the spatial distributions shown in Figure 2 are binned for substorm phase. In order to determine the phase of a substorm that a given measurement corresponds to, the Substorm Onsets and Phases from Indices of the Electrojet (SOPHIE) technique (Forsyth et al., 2015) is applied to the SuperMAG AL index (Gjerloev, 2012; Newell & Gjerloev, 2011), using an expansion percentile threshold of 75. This technique involves evaluating the rate of change of the SuperMAG AL index with 1‐min time resolution. A negative rate of change below the threshold is identified as an expansion phase and a positive rate of change above the threshold is identified as a recovery phase. All other times are identified as growth phases. For full details on the SOPHIE technique, the reader is referred to Forsyth et al. (2015). For the data sets used in this study, the SOPHIE technique identifies 8,142 substorm events during the time period considered. Unique sampling of 5,099 growth phases, 5,833 expansion phases, and 6,214 recovery phases are provided. The corresponding median (mean) phase durations are 90 (211), 21 (26), and 36 (44) min for the growth phase, expansion phase, and recovery phase, respectively. These typical phase durations are in good agreement with the expected values (Forsyth et al., 2015).

It is noted here that we consider an average over all measurements within a given substorm phase and independent of storm time conditions. Although it is well established that the ring current demonstrates significant dependences on storm activity, in order to isolate and examine substorm‐associated variations we only consider comparisons with substorm phase. Therefore, this analysis is independent of storm time and the results correspond to an average over both quiet time and storm time substorms. However, future work will aim to explore the substorm contributions to the ring current in further detail by assessing dependences on storm time conditions.

**Figure 4:**
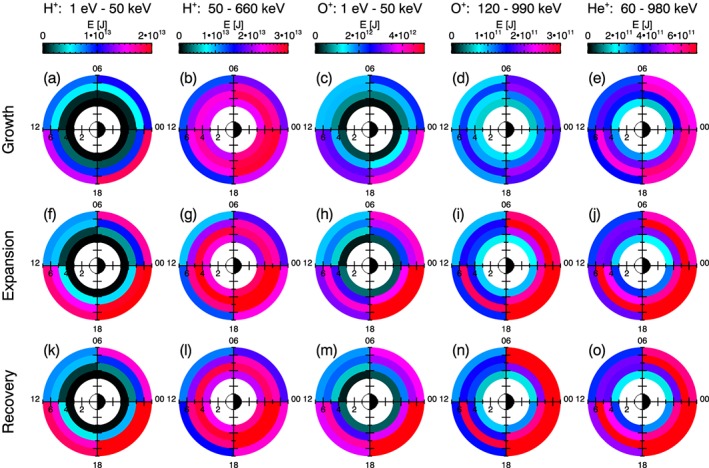
Median energy, E, [J] for each L‐magnetic local time bin is plotted at the bin's location in the L‐magnetic local time domain for each ion data set, as labeled. Observations are binned for substorm phase, showing values during the growth phase (a)–(e), expansion phase (f)–(j), and recovery phase (k)–(o). Substorm phases are identified according to the Substorm Onsets and Phases from Indices of the Electrojet algorithm (Forsyth et al., 2015).

The energy values are binned for growth phase, expansion phase, and recovery phase, and the resulting distributions are shown in Figure 4. Figures 4a–4e show the median energy values for each *L*‐MLT bin in the magnetic equatorial plane during the growth phase for each ion data set, as labeled. In the same format, Figures 4f–4j and Figures 4k–4o show the median energy values for the expansion phase and recovery phase, respectively.

Initial visual inspections indicate an increase in energy content after substorm onset for all ion data sets, with the most significant change occurring between the growth phase and the expansion phase rather than the expansion phase and the recovery phase. To provide an indication of the statistical significance of the energies shown in Figure 4, the reader is referred to Figure S2, where the corresponding number of spacecraft passes through each spatial bin are shown. It can be seen that there are a sufficiently large number of energy samples for each spatial bin in a given substorm phase (typically ∼10^2^) for reliable statistics.

**Figure 5:**
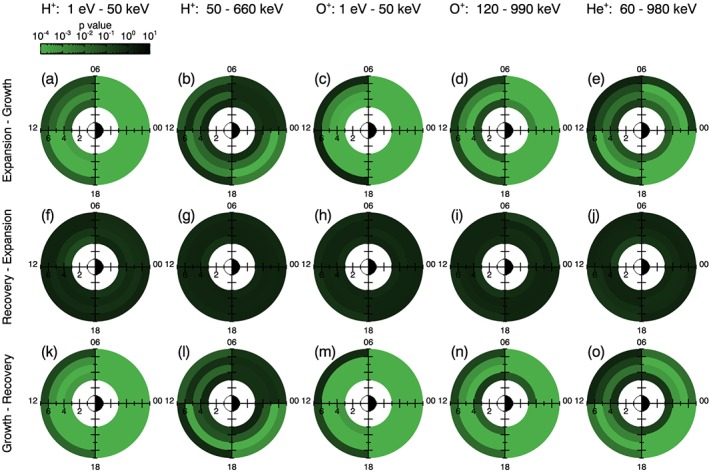
Energy distributions in each L‐magnetic local time bin are compared for different substorm phases using the Kolmogorov‐Smirnov test. The p value of the test is plotted for each L‐magnetic local time bin, and results are shown for each ion data set. The comparison between growth and expansion phase (a)–(e), expansion and recovery phase (f)–(j), and growth and recovery phase (k)–(o) is shown.

In order to assess how the energy varies with substorm phase the Kolmogorov‐Smirnov test is utilized. The distribution of energy values in a given *L*‐MLT bin and for a given ion data set are compared for two different phases. The Kolmogorov‐Smirnov test is applied to obtain a *p* value, representing how similar the two distributions are, and the results are shown in Figure 5. Figures 5a–5e compare the growth phase and the expansion phase, Figures 5f–5j compare the expansion phase and the recovery phase, and Figures 5k–5o compare the growth phase and the recovery phase. Values lower than a typically defined threshold of 0.01 suggest that the means of the energy distribution are statistically different and that a statistically significant change in energy is present for the different substorm phases. We note that although the Kolmogorov‐Smirnov test is presented here, the use of alternative statistical tests (e.g., the Mann‐Whitney‐Wilcoxon test or the Student's *T* test) do not alter the results. We have opted to use the Kolmogorov‐Smirnov test in this analysis as it is distribution free, and therefore does not require any prior assumptions about the distribution of energy values to be made.

Figure 5 shows that the lowest *p* values are generally present in the comparison of the expansion phase to the growth phase, as well as the recovery phase compared to the growth phase. In contrast, relatively high values above the 0.01 threshold are observed for all *L*‐MLT sectors and all ion data sets when comparing the recovery phase to the expansion phase. This suggests that there is a significant change in energy from the growth phase to the expansion phase, followed by no significant change from the expansion phase to the recovery phase. The energy in the recovery phase remains statistically different to the original growth phase energy distributions.

Figure 5 also demonstrates *L*‐MLT variations, with *p* values having the lowest values in the 18–24 MLT sector. This shows that the most significant changes occur in the dusk‐midnight sector. Furthermore, the lowest *p* values are observed for the low energy H^+^ ions and the O^+^ ions, compared to the other ion data sets. The processes that may drive these observations are discussed later.

**Figure 6:**
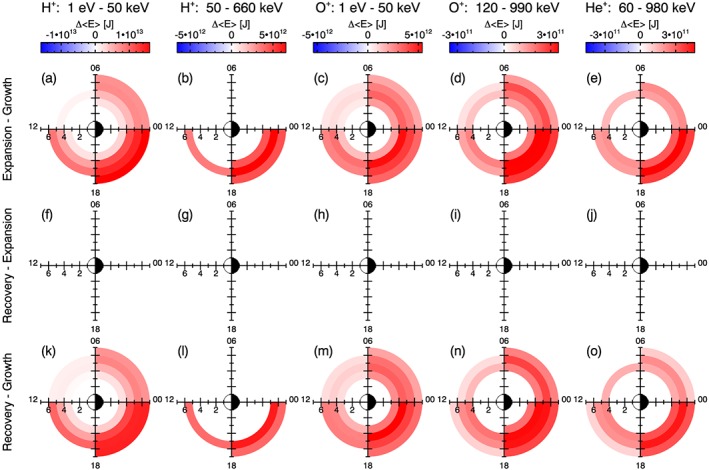
In the same format as Figure 5, the difference in mean energy, Δ<E>, [J] for a given L‐magnetic local time bin between two substorm phases is plotted, considering each ion data set separately. Note that the value is only plotted if the corresponding p value for the bin is less than 0.01. The color scale is such that red indicates an increase in energy with phase and blue indicates a decrease in energy.

For *L*‐MLT bins that were identified to have statistically significant differences in mean energies between two substorm phases (*p* < 0.01), the difference in the mean energies are shown in Figure 6. The color scale is such that increases in energy with substorm phase are colored red, and decreases in energy are colored blue. If a given bin does not have statistically different mean energy values (i.e., *p* ≥ 0.01), the bin is not plotted in Figure 6. Figure 6 clearly demonstrates the location, as well as the magnitude of, increases and decreases in average energy in *L* ‐ MLT space over the course of a substorm, for all ion sets considered here.

Figure 6 shows that the average energy is increased over almost all *L*‐MLT bins over all ion data sets for the expansion phase compared to the growth phase. The magnitude of the increase is largest in the 18–24 MLT sector and tends to increase with increasing *L* value for this MLT sector.

The different ion data sets indicate differences in the local time distribution, when comparing the expansion phase to the growth phase (Figures 6a–6e). Whereas the high‐energy protons show enhancements in the energy content restricted to the midnight to noon MLT sectors (Figure 6b), the other ion data sets show enhancements in energy observed across all local time sectors. For the other ion data sets, no decreases in the energy content are observed for any of the *L*‐MLT bins.

Figures 6f–6j show that there is no statistically significant change in the energy content between the expansion phase and recovery phase distributions, as no values have been plotted. Correspondingly, the general features of the *L*‐MLT distributions for a given data set appear to differ little between the expansion phase and recovery phase, when compared to the growth phase distribution (comparing Figures 6a–6d to Figure 6k–6o).

**Figure 7:**
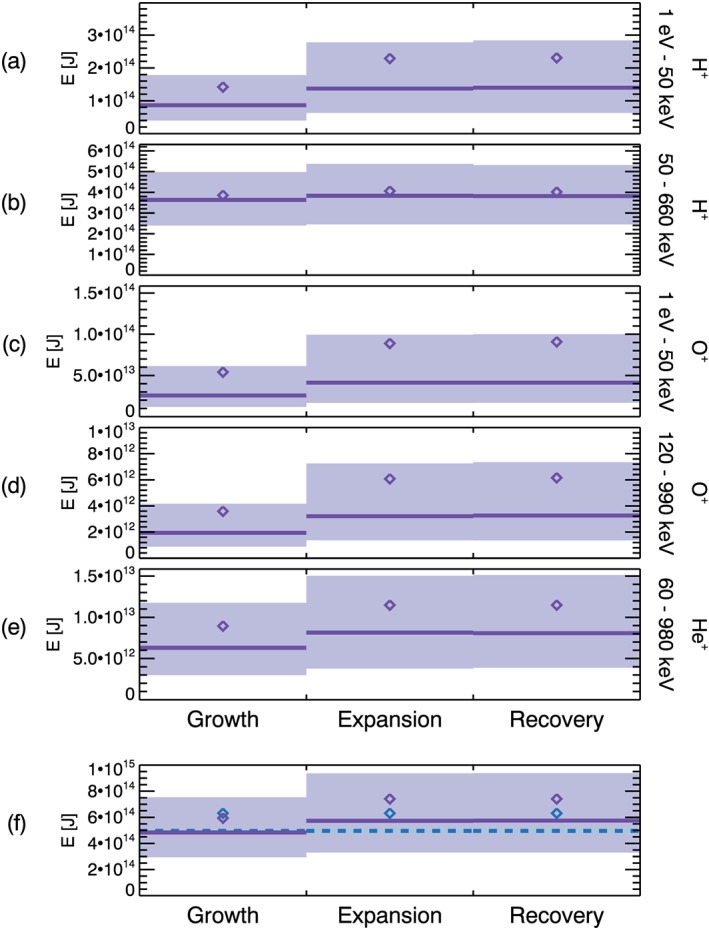
The purple lines in (a)–(e) show the sum of the median energies, E, [J] over all L‐MLT bins for each substorm phase (growth, expansion, and recovery), where each panel corresponds to a different ion data set. The sum of the mean energies are shown by the purple diamonds. The sum of the lower quartile values and the upper quartile values over all L‐MLT bins are represented by the lower and upper limits of the light purple shaded regions. (f) shows the sum over all ion data sets for each substorm phase, equivalent to the sum of the values shown in (a)–(e), where the purple lines correspond to the median values, the purple diamonds correspond to the mean values, and the light purple shaded regions correspond to the extent of the lower quartile to the upper quartile values. The total energy value summed over all L‐MLT and all ion data sets for all times, corresponding to values shown in Figure 3, is indicated by the blue dashed line. The mean value from the data shown in Figure 3 is also shown by the blue diamonds. MLT = magnetic local time.

Using the median energies shown in Figure 4, the global energy content of the ring current for each substorm phase and each ion data set can be estimated by summing over all *L*‐MLT bins, as done for the average ring current values (see Figure 3). The results are shown in Figures 7a–7e, where each panel corresponds to a different ion data set, as labeled. For each panel, the sum of the median values over all *L*‐MLT bins are shown as the purple lines for each substorm phase, and corresponding sum of the mean values are indicated by the purple diamonds. The lower limit of the light purple shaded region represents the sum of the lower quartile values and the upper limit of the light purple shaded region corresponds to the upper quartile values. Changes in the global median energy content for each data set with substorm phase are apparent. Comparing the growth phase to the expansion phase demonstrates an increase in the average global energy content by more than 50% for given ion data sets. Specifically, Figure 7a shows that the low energy H^+^ ion contribution increases from 9 × 10^13^ to 1.4 × 10^14^ J over substorm onset. This is also true of the low energy O^+^ increasing from 3 × 10^13^ to 4 × 10^13^ J (Figure 7c) and the high‐energy O^+^ increasing from 2 × 10^12^ to 3 × 10^12^ J (Figure 7d). Relatively modest increases are observed in the global energy content for the high‐energy H^+^, where an increase from 3.6 × 10^14^ to 3.8 × 10^14^ J occurs over onset (Figure 7b) and for He^+^, showing an increase from 6 × 10^12^ to 8 × 10^12^ J (Figure 7e). This demonstrates a clear dependency on ion species and ion energy for the substorm energization of the ring current. It is also noted that the shape of the energy distribution appears to remain relatively constant across the substorm process. As the ring current will include the contributions for all ions over all energies, the global energy content of the ring current can be estimated by summing the values shown in Figures 7a–7e over all ion data sets, and the results are shown in Figure 7f in the same format as the other panels. For comparison, the blue dashed line indicates the total median energy summed over all ion data sets for the total data set prior to substorm phase binning (corresponding to Figure 3), representing the average global energy content for typical magnetospheric conditions. The corresponding mean value is also included and is shown by the blue diamonds.

As identified previously, an increase in the ring current energy content is observed for the expansion phase compared to the growth phase for all ion data sets (Figures 7a–7e). The energy content remains enhanced in the recovery phase. The shaded region indicates the estimated upper and lower quartiles of the energy content, with Figures 7a–7e demonstrating a large spread in values relative to the energy change following substorm onset. By summing over all ion data sets, the total energy content of the ring current for each substorm phase is estimated and the results are shown in Figure 7f. For context, the blue dashed line shows the total energy content calculated from the full data set over all conditions, therefore representing typical magnetospheric conditions and the average energy of the ring current. Figure 7f shows that the growth phase value and the average value (blue dashed line) are in very close agreement, as expected. The increase in total energy content from the growth phase to the expansion phase is calculated as 0.9 × 10^14^ J.

## 5 Discussion

### 5.1 Average Energy Content of the Ring Current

The analysis presented here has identified some key features of the typical ring current based on an average of ion measurements between 2012 and 2017. In good agreement with previous results (e.g., Krimigis et al., 1985), the main contributor to the ring current energy content is protons within the energy range of ∼50–660 keV, as shown by Figures 2a–2e and 3. The energy content of the high‐energy protons represents approximately 73% of the total energy content. In addition, local time variations in the energy content are apparent from Figures 2a–2e, where values are peaked in the premidnight MLT sector for all ions and energy ranges considered here. This distribution is consistent with the westward drift of positively charged particles. As particles are convected into the ring current region from the nightside plasma sheet, they will drift into the dusk‐midnight sector, so the density will be the greatest in this region. Loss processes, due to convection of ions on open drift paths to the dayside magnetopause (Liemohn et al., 1999, 2001; S. Takahashi et al., 1990), charge exchange (Dessler & Parker, 1959; Fok et al., 1995; Jordanova et al., 1996; Stuart, 1959), and wave‐particle interactions (Cornwall, 1965; Russell et al., 1970; Southwood et al., 1969), act to increasingly reduce the density in the succeeding sectors moving clockwise in MLT.

Based on an average of energy content measurements over the full data set, the typical ring current energy is estimated as 5.0 × 10^14^ J. This measurement is based on a period spanning 2012–2017, and correspondingly is representative of the magnetospheric conditions around the maximum of the solar cycle. As the occurrence of storms and level of geomagnetic activity are known to vary with solar cycle (Gonzalez et al., 1990; Legrand & Simon, 1991; Sugiura, 1980), the estimated average energy content is also expected to vary. The dependence of the average energy content on solar cycle is an area of future analysis.

By applying the average energy content (5.0 × 10^14^ J) to the DPS relationship, we estimate that the ring current typically provides a magnetic field perturbation of −12.4 nT. This value is in good agreement with observed values of the Dst index. Specifically, O'Brien and McPherron (2000) observed a quiet time Dst index value of −11 nT on average, and we have calculated the mean SYM‐H index value during the time period spanned by the data set used here as −12 nT.

### 5.2 How Does the Ring Current Energy Content Vary With Substorm Phase?

This study also explores how the energy content varies, on average, with substorm phase. As identified in the previous section, the energy content shows several differences in spatial distribution and magnitude with substorm phase, and the implications of these variations will now be discussed. Figures 4 and 6a–6e show that, for all ion data sets, the energy content is increased following substorm onset. The increase in energy is observed to be the largest in the 18–24 MLT sector. This observed feature supports the process of ion injection at substorm onset from the magnetotail, where the ions then drift duskward (Lopez et al., 1990; Mauk & McIlwain, 1974; McIlwain, 1974; Reeves et al., 1990). Furthermore, a larger increase is observed in the premidnight sector compared to the postmidnight sector. This is in agreement with previous work that has shown substorm injections of ions have an asymmetric occurrence distribution, such that they are more likely to occur in the premidnight MLT sector compared to the postmidnight MLT sector (Birn et al., 1997; Gabrielse et al., 2014; Sarris et al., 1976; Thomsen et al., 2001).

Investigating the local time dependence further provides additional features of interest. Figure 6b shows that the high‐energy protons are enhanced for the premidnight sector and the afternoon sector, however, the enhancement does not extend into the following local times. Comparing the drift time of a proton in the high‐energy range (∼20 min, Schulz & Lanzerotti, 1974) with the mean expansion phase duration (21 min, Forsyth et al., 2015), it would be expected that the injected high‐energy protons have drifted across all MLT sectors, and therefore, the energy content is increased across all local times. The lack of an enhancement in the morning and postmidnight MLT sectors suggests a loss of ions with energies within 50–660 keV before a full drift period has been completed. In contrast, the low energy protons demonstrate an increase in energy over all MLT (Figure 6a). These low energy protons will have a longer drift period (∼6 hr, Schulz & Lanzerotti, 1974) compared to the high‐energy protons, so it is not expected that injected ions in the energy range will drift across all MLT sectors within the expansion phase. It can be inferred that the increase in energy content is therefore due to the transfer of flux in the high‐energy band to the low energy band as the protons lose energy over their drift path.

The possible cause of energy loss for a drifting proton can be wave‐particle interactions, in particular drift‐bounce resonance. The substorm‐injected westward drifting ion population can act as a source of energy for the excitation of plasma waves due to nonequilibrium phase space particle distributions. In particular, drift‐bounce resonance of protons can drive high‐m Pc5 pulsations (James et al., 2013; Southwood, 1976; K. Takahashi et al., 1985; Woch et al., 1990; Yeoman & Wright, 2001), which has been previously observed to occur predominantly in the afternoon MLT sector (Anderson et al., 1990; Woch et al., 1990). The transfer of energy from the proton population to the waves would result in a decrease in the flux of particles in the high‐energy range and increase in flux for the lower energy range. Furthermore, resonances with Electromagnetic Ion Cyclotron (EMIC) waves would also act to de‐energize the protons. EMIC wave generation is associated with thermally anisotropic ion populations and can maximize in regions where energetic and cold dense plasma populations overlap (Mauk & McPherron, 1980; Morley et al., 2009). Studies have observed EMIC wave excitation in the H‐band to occur most frequently in the afternoon sector and close to the magnetic equator, where hot westward drifting ring current particles injected from the nightside plasma sheet and cold plasmaspheric populations coexist (Allen et al., 2015; Clausen et al., 2011; Cornwall, 1965; Erlandson & Ukhorskiy, 2001; Keika, Takahashi, et al., 2013; Remya et al., 2018). Through cyclotron resonant interactions with EMIC waves, injected high‐energy protons could be effectively de‐energized during their drift trajectory in the noon‐dusk MLT sector, such that flux is transferred from the high‐energy band to the low energy band and resulting in the observed features shown in Figures 6a and 6b.

A change in drift trajectories could also produce the observed changes in energy content for the high‐energy protons (Figure 6b). For a particle injected from the nightside plasma sheet, the trajectory extends to higher *L* values for increased particle energy and the number of protons on open drift paths is increased (Cowley & Ashour‐Abdalla, 1976; Ozeke & Mann, 2001). Therefore, particles with higher energies are lost to the magnetopause and would not populate the local time sectors westward of 12 MLT. At substorm onset, the energy distribution of protons within the 50–660 keV band may have changed, such that there are more higher energy particles. This change would act to increase the number of protons that are located on open drift paths and are subsequently lost to the magnetopause. Future analysis will examine the variation in energy distributions for protons during the substorm process, hence providing insight into how the drift trajectories of the protons change.

Alternatively, the movement of the magnetopause to lower radial distances could contribute to the decrease in flux for the high‐energy protons through increasing the number of open drift trajectories. Based on a review of previous literature, it has not been clearly established as to how the magnetopause location varies following substorm onset. Although previous work has identified that the occurrence of southward directed IMF during the substorm growth phase results in the inward motion of the magnetopause (Aubry et al., 1970; Meng, 1970), the response of the magnetopause position following substorm onset in the expansion phase has not been directly examined by previous studies. If the magnetopause continues to move to lower radial distances on the dayside, the magnetopause could intersect particle trajectories that were previously on closed drift paths, and increased loss of ring current particles to the magnetopause could occur. Previous studies have shown that, on average, substorms are associated with continued southward directed IMF after onset, which is expected to result in continued erosion of the dayside magnetopause (Forsyth et al., 2015; Milan et al., 2009; Suvorova et al., 1999; Walach & Milan, 2015). However, the variation in magnetopause position is not fully understood and requires further analysis in order to establish the role of magnetopause movements in the loss of ring current ions.

Unlike the high‐energy protons, enhancements in energy content across all local times are observed for the O^+^ and He^+^ ions (Figures 6c–6e). The heavy ions have longer lifetimes than H^+^ ions (Smith et al., 1981), and therefore, can complete several drift periods before being energized. However, a decrease in the magnitude of the energy enhancement is observed for both the O^+^ ions and He^+^ ions, comparing the dusk‐midnight MLT sector to the noon‐dusk MLT sector. This suggests a decrease in the flux of these ions within their respective energy bands, as they drift westward from the nightside injection region. Similarly to the proton cyclotron resonance described previously, ion cyclotron resonance can also drive EMIC waves in the He‐band and O‐band, with occurrence peaking in the afternoon MLT sector (Keika, Kistler, et al., 2013; Min et al., 2012; Yu et al., 2015). The EMIC wave interaction allows for a transfer of energy from the He^+^ and O^+^ ions, thus resulting in a decrease of energetic ion flux as they drift westward through the afternoon MLT sector.

The global variation in energy content is shown by Figure 7, which presents several features of interest. By summing over all ion contributions, the increase in total energy content of the ring current is estimated as 0.9 × 10^14^ J. This is an increase of ∼19% of the average energy content in the growth phase. Compared to the typical 10^15^ J of energy released in a substorm (Tanskanen et al., 2002), the increase in ring current energy accounts for approximately 9% of the total substorm energy budget. This provides a statistical quantification of the substorm energy input to the ring current, with the results showing a nonnegligible input.

The analysis presented here has estimated the global energy content of the ring current as contributed by the ion populations. Although the contribution of electrons is expected to be insignificant due to their negligible energy density compared to ions (Baumjohann, 1993), Liu et al. (2005) suggested that the contribution of electrons may be important during storm time conditions. However, this is not expected to notably impact the results shown here.

It is also noteworthy to compare the increase in energy for the expansion phase compared to the growth phase for the different ion data sets. Although the majority of the ring current energy is contributed by the high‐energy protons (Figures 4 and 7), the change in energy is significant for the heavy ions. It can be seen from Figures 4 and 7 that the energy content increases by ∼100% for the O^+^ ions, in comparison to a more modest relative increase for the high‐energy protons. This result is in agreement with multiple previous studies (Balsiger et al., 1980; Daglis et al., 1993; Daglis & Axford, 1996; Fu et al., 2002; Hall et al., 1998; Kistler et al., 1990; Lennartsson et al., 1985; Sandhu et al., 2016; Young et al., 1982), where enhanced heavy ion outflows at high latitudes result in an increase in the heavy ion concentration in the tail plasma sheet. Consequently, the plasma injected into the inner magnetosphere has a relatively increased concentration of O^+^, and significantly increases the flux of heavy ions in the ring current. Furthermore, Keika, Kistler, et al. (2013) showed that impulsive electric fields act to accelerate heavy ions more effectively compared to light ions, due to their larger gyroperiod allowing for more effective adiabatic heating.

Overall, it has been shown that there is a statistically significant increase in the ring current energy content during the substorm process. However, there are some implications to averaging over the multiple substorms identified by the SOPHIE algorithm. Previous work has shown that substorms are a highly variable phenomenon, with not all substorms being observed to be associated with an injection of energetic particles into the ring current (Abel et al., 2006; Boakes et al., 2009, 2011). In particular, Boakes et al. (2011) observed that substorms are associated with varied injection signatures, with only 33% of substorm events associated with a classical injection signature at geosynchronous orbit. This suggests that not all substorms will drive an injection of energized plasma into the inner magnetosphere, and therefore, not all substorms will drive enhancements in the ring current energy content. Furthermore, this analysis does not differentiate between isolated substorms compared to sequences of closely spaced substorms. The approach also combines substorms during both storm times and nonstorm times. Consequently, the increase in energy demonstrated in Figure 7 represents an increase at substorm onset averaged over varied ring current conditions and responses. Future work will aim to understand how the energy input into the ring current during a substorm varies, in the context of previous findings by Wang et al. (2003) and Boakes et al. (2011) who showed that the occurrence and strength of a substorm‐associated injection is dependent on the solar wind driving conditions.

## 6 Summary and Conclusions

To conclude, this study has used RBSPICE and HOPE measurements combined with a substorm event list identified by the SOPHIE technique to conduct a statistical analysis of how the ring current energy content varies with substorm phase. During the substorm process, a statistically significant enhancement in the ring current energy is observed following substorm onset in the expansion phase, with the energy enhancement persisting into the recovery phase. The median energy input into the ring current is estimated as ∼0.9 × 10^14^ J, which represents ∼9% of the average energy released in a substorm.

It has also been observed that the energy content of the ring varies strongly with local time, with the largest energy content contained within the dusk‐midnight MLT sector. Following substorm onset, the magnitude of the increase in energy is greatest in the premidnight MLT sector, extending westward, with features indicative of a substorm‐associated injection of energetic ions convected from the tail plasma sheet. Furthermore, a consideration of the expected gradient‐curvature drift periods of the ions, as well as a comparison of the different ion sets, suggest a loss of energetic plasma through wave‐particle interactions in the afternoon MLT sector.

A strong relative enhancement in energy content for the O^+^ ions following substorm onset was observed. This is expected to result from enhanced heavy ion outflows from the ionosphere, increasing the heavy ion concentration of the plasma sheet, and therefore contributing to O^+^‐rich plasma injections into the ring current region. This is a noteworthy result, in support of substorms being an important factor in producing the O^+^‐rich ring current associated with storm conditions (C:son Brandt et al., 2003; Daglis, 1997; Fu et al., 2002; Hamilton et al., 1988; Mitchell et al., 2003; Ohtani et al., 2005).

Overall, an investigation of the ring current energy has shown that the substorm process contributes a nonnegligible amount of energy following substorm onset. Understanding the sources of variability in the ring current enhancement is an area of future work, and will provide an insight into how the substorm process is coupled to the ring current properties.

## Supporting information



Supporting Information S1

Figure S1

Figure S2
