# Supplementary figures and images for: Energization of the Ring Current by Substorms
Source: J Geophys Res Space Phys. 2018 Oct 8;123(10):8131–48. doi: 10.1029/2018JA025766 (PMC6360953; doi:10.1029/2018JA025766)

H<sup>+</sup>: 1 eV - 50 keV    H<sup>+</sup>: 50 - 660 keV    O<sup>+</sup>: 1 eV - 50 keV    O<sup>+</sup>: 120 - 990 keV    He<sup>+</sup>: 60 - 980 keV

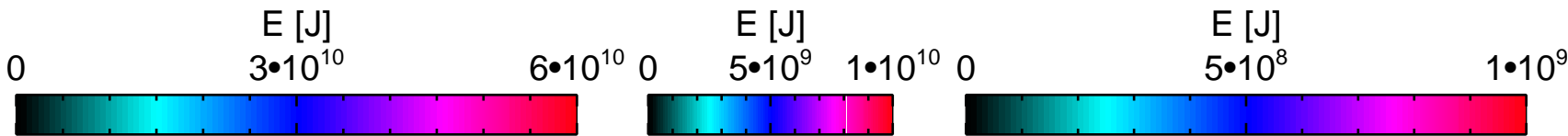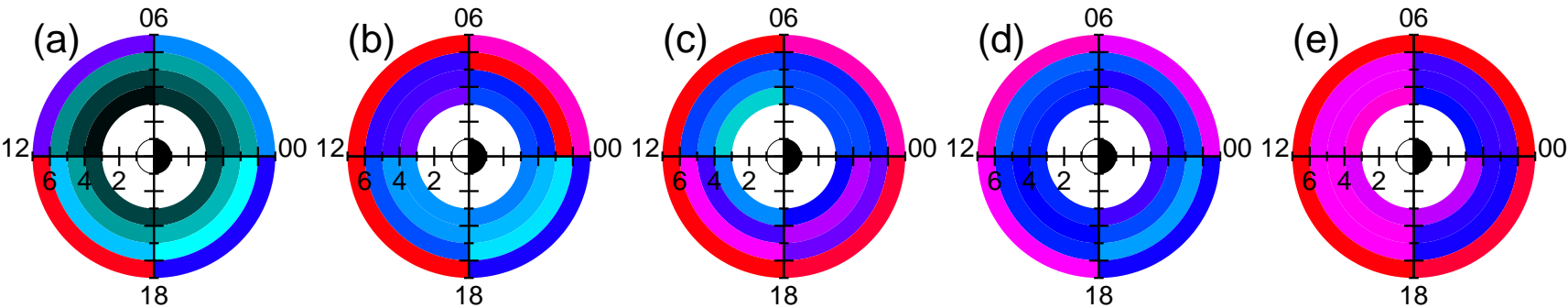

Supplement: Supplementary file 2 — Figure S1 [file JGRA-123-8131-s002.pdf]

H<sup>+</sup>: 1 eV - 50 keV

H<sup>+</sup>: 50 - 660 keV

O<sup>+</sup>: 1 eV - 50 keV

O<sup>+</sup>: 120 - 990 keV

He<sup>+</sup>: 60 - 980 keV

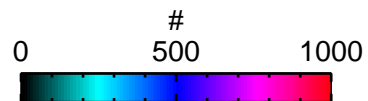

Growth

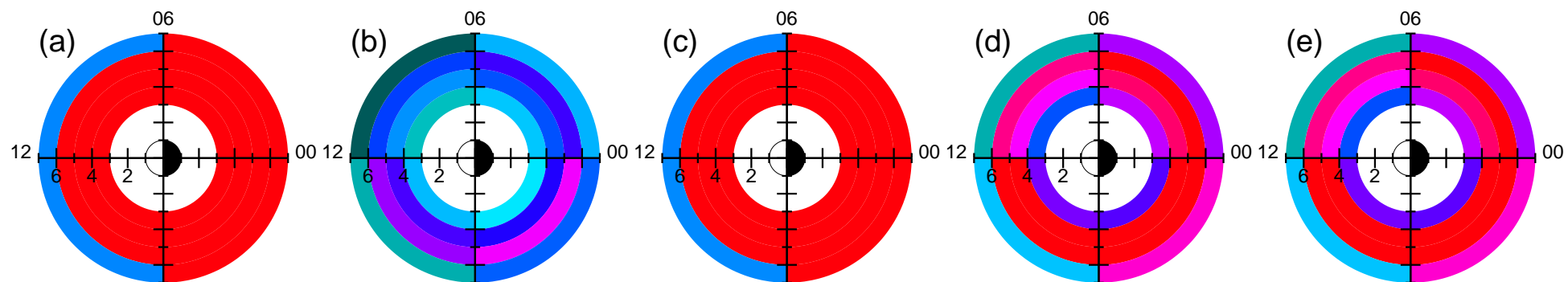

Expansion

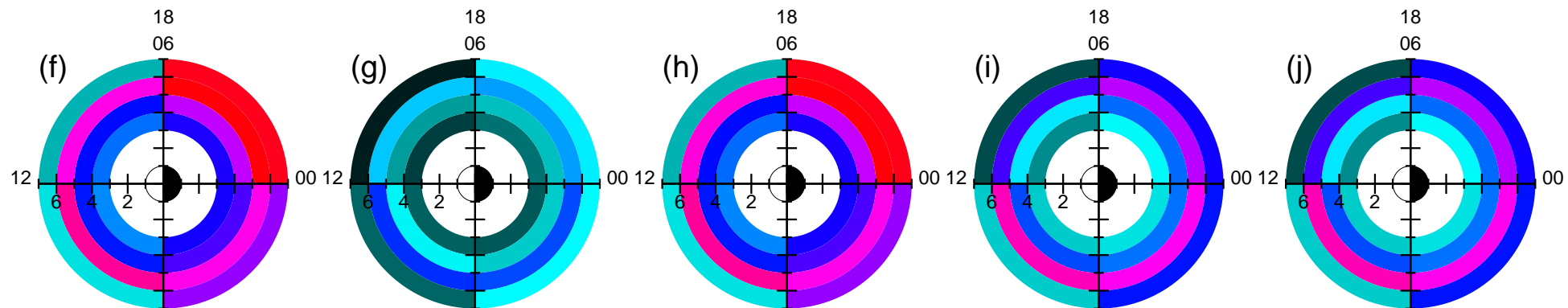

Recovery

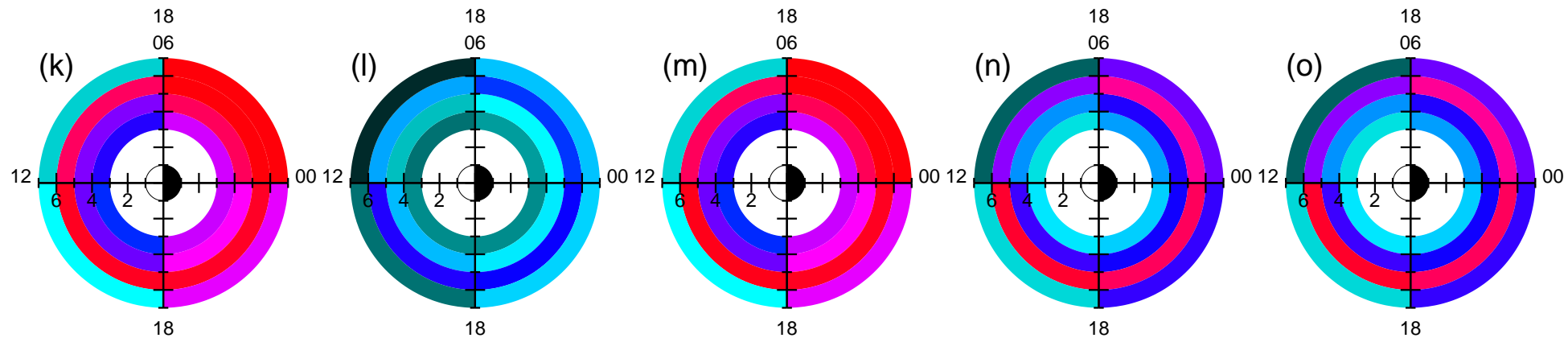

Supplement: Supplementary file 3 — Figure S2 [file JGRA-123-8131-s003.pdf]
